# Streamlining an existing hip fracture patient pathway in an acute tertiary adult Irish hospital to improve patient experience and outcomes

**DOI:** 10.1093/intqhc/mzz093

**Published:** 2019-12-22

**Authors:** Caitriona Murphy, Eithne Mullen, Karrie Hogan, ronan O’toole, Seán Paul Teeling

**Affiliations:** 1 Physiotherapy Department, Mater Misericordiae University Hospital, Eccles Street, Dublin 7, Ireland; 2 Service Improvement Team, Ireland East Hospital Group, c/o Mater Misericordiae University Hospital, Eccles Street, Dublin 7, Ireland; 3 Cancer and Surgery Directorate, Mater Misericordiae University Hospital, Eccles Street, Dublin 7, Ireland; 4 Department of Medicine for the Elderly, University Hospital Limerick, Dooradoyle, Limerick, Ireland; 5 School of Nursing, Midwifery and Health Sciences, University College Dublin, Belfield, Dublin 4, Ireland

**Keywords:** hip fracture care, process improvement, Lean Six Sigma, interdisciplinary working, integrated care pathways, healthcare outcomes

## Abstract

**Objective:**

To improve access for hip fracture patients to surgery within 48 h of presentation to the Emergency Department, and to increase the number of patients receiving pre-operative orthogeriatric review, through streamlining an existing hip fracture patient pathway.

**Design:**

A pre–post design involving a multi-disciplinary team use of the Define, Measure, Analyse, Improve and Control framework integral to Lean Six Sigma (LSS) methodology, to assess and adapt the existing hip fracture pathway from presentation to Emergency Department to the initiation of surgery.

**Setting:**

A 600-bed teaching hospital in Ireland.

**Participants:**

Nursing, medical, administrative and physiotherapy staff working across Emergency Medicine, Orthogeriatrics and Orthopaedic Specialities and Project management.

**Interventions:**

LSS methodology was used to redesign an existing pathway, improving patient access to ortho-geriatrician assessment, pain relief and surgery in line with the Irish Hip Fracture Data Base Key performance indicators.

**Main Outcome Measures:**

Access to pain relief, access to surgery and volume of patients receiving ortho-geriatric assessment.

**Results:**

The percentage of patients undergoing surgery within 48 h of presentation to Emergency Department increased from 55% to 79% at 3 months, and to 85% at 6 months. Improvements were also achieved in the secondary performance metrics relevant to quality of patient care. All care pathway changes were cost neutral.

**Conclusions:**

Hip fracture surgery within 48 h of presentation to hospital is a recognized standard of hip fracture care associated with decreased length of stay and decreased mortality. With respect to this performance metric, this intervention has contributed to improved patient outcomes.

## Introduction

Hip fractures have long been recognized as a substantial public health problem in western countries [1]. Increased time to surgery for hip fracture patients is associated with both increased length of stay and increased mortality [2,3]. Hip fracture surgery within 48 h of presentation to hospital is a recognized key standard of hip fracture care that is relevant both to quality of patient care and financially efficient care [4]. Following the identification of suboptimal performance relative to such standards, the prime objective of this study was to use Lean Six Sigma (LSS) methodology to identify and implement process improvements in the hip fracture clinical pathway in an acute hospital in Ireland.

LSS methodology, a process improvement approach originating in the manufacturing sector, has been adopted by healthcare providers to improve efficiency, safety and patient satisfaction in healthcare delivery [5]. Lean is focused on improving flow and eliminating waste for the customer/patient; it is intended to respond to the needs of patients through operating a ‘customer pull’ [6,7]. Six Sigma (a term developed by Motorola) involves seeking perfection through the elimination of variability [ 8]. This project uses LSS, a combined approach working towards improved flow and efficiency [9].

Between 2008 and 2013, consequent to the global economic crisis, national budget reductions in Health of €3.3bn (22%) were implemented in Ireland [10]. Healthcare service providers in Ireland, and indeed elsewhere, have been challenged to maintain and improve services and patient care whilst operating under financial constraint. There is a requirement for effective, high-quality person-centred care that is efficient and cost effective.

Drotz and Poksinski [11] suggest that much of the published literature pertaining to the use of LSS in healthcare neglects to expand on the implementation process and associated changes in task distribution, changes in role, teamwork and leadership, with preference for an emphasis on the before and after effects of process change. A theme also noted amongst the literature is the need for ‘Lean Thinking’ to become a cultural movement or ‘organisational philosophy’, requiring both a ‘top down’ and ‘bottom up’ approach within organisations [5,7,12,13].

This paper contributes to the growing body of published evidence that LSS methodology can be successfully employed to optimize care and resource use in the health sector, and will expand on the key changes that facilitated improvements in process and patient-centred care.

## Methods

### Project team

The multidisciplinary LSS project team was assembled, consisting of nursing, medical, administrative and physiotherapy staff working across Emergency Medicine, Orthogeriatrics and Orthopaedic Specialties, as well as Project management, in a large acute, teaching hospital in Ireland.

The project was undertaken using the Define, Measure, Analyse, Improve and Control (DMAIC) method integral to LSS methodology, resulting in a sequential five step process, namely define, measure, analyse, improve and control phases, a combination of project management and business process improvement techniques [14]. These individual but interdependent stages will each be outlined to illustrate the methodology used.

### Initial assessment

#### Define

The define phase of a project allows the LSS team to identify the correct and precise objective of a project, a fundamental in Lean methodology [15]. Each project requires a ‘project charter’, with clearly documented project justification and objectives; the charter also defines the stakeholders and team members. The charter’s purpose is to provide project focus, but it is not a solution finding exercise; it is important that project goals align with the organisational philosophy and strategic organisational goals [16].

Evaluating the initial project charter utilising a specific, measurable, attainable, realistic, and timely analysis allowed the project scope to be more clearly outlined, and relevant and achievable goals to be identified. Critical to quality analysis linked the goals to defined metrics to facilitate assessment of progress and effect [17]. Given the adverse consequences of delayed surgery for hip fracture patients, the prime performance metric used in this project was the percentage of patients having surgery (induction of anaesthesia) within 48 h of presentation to the hospital’s Emergency Department.

Two further performance metrics relevant to quality of patient care were used. First, the percentage of patients receiving orthogeriatric pre-operative review was examined, as access to orthogeriatric care is one of six key standards of hip fragility fracture care identified by the British Orthopaedic Association [4].

A secondary performance metric that had not been identified in the initial project scope, namely the percentage of patients receiving optimal analgesia (fascia iliaca nerve block) pre-surgery, was added at the specific request of one of the key stakeholders, the Department of Anaesthesia. They identified this as a patient-centred performance metric that improves patient experience through earlier effective pain control, but also leads to more streamlined care once the patient enters the surgical theatre, as patients with effective pain control can be more easily and efficiently prepped for surgery.

**Figure 1:**
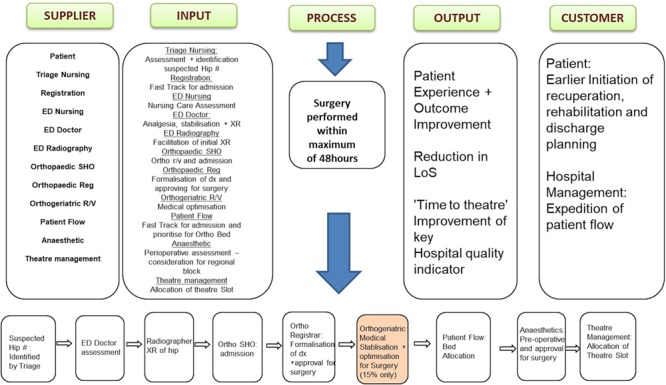
SIPOC identifying the key stakeholders and their role in the hip fracture patient pathway.

The introduction of this secondary metric at the request of a key stakeholder highlighted the importance of stakeholder identification and engagement and the necessity of developing a communication plan for the relevant parties. This was achieved by completing Supplier, Input, Process, Output, Customer (SIPOC) mapping, a first step in identifying those involved in the process and their role (Fig. 1) [18].

The project charter’s stated aim was to improve the hospital’s performance with respect to the key performance metrics from baseline and to exceed the Irish national averages for each outcome assessed.

#### Measure

A focused data collection plan was formulated and co-ordinated by the LSS team, to ensure all relevant data were available for analysis [19] to allow understanding of the current state [20]. Current process performance was examined and measured in several different ways.

In Ireland, each hospital submits hip fracture performance metrics to the National Office of Clinical Audit, which is published in the Irish National Hip Fracture Database (IHFD). The hospital’s IHFD data for 2014, the year prior to the Lean project, was used as the baseline measure of the hospital’s performance in hip fracture care. This dataset includes all patients ≥65 years that presented to the hospital with a hip fracture in that year. The Irish National averages for each performance metric were sourced from the IHFD National Report 2014 [21].

Understanding the ‘Current State’ or the experience of hip fracture patients presenting to the hospital was essential. This was achieved by ‘Going to Gemba’, an observational method of current process for gathering data [22]. One real-time Gemba visit was completed with project team members physically following and tracking the journey of a single patient from arrival to ED until they were brought to theatre for surgery. Four retrospective chart audits were completed via review of patient electronic and paper records.

**Figure 2:**
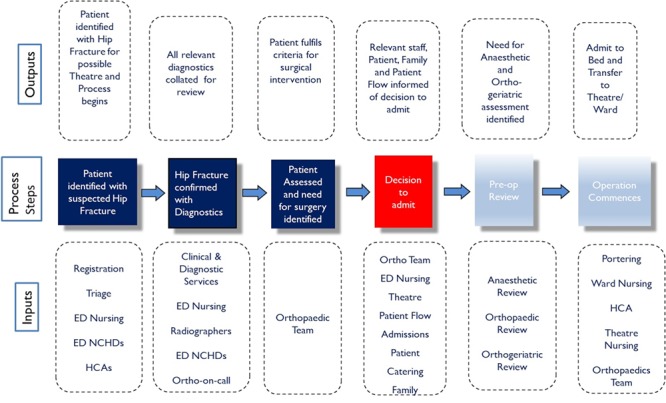
Measure phase IPO process map depicting ‘current state’ hip fracture pathway.

Value stream mapping, or process mapping, is a means by which steps in a process are visually mapped [23]. The information collated regarding the process was visually illustrated using a high level Input Process Output (IPO) map (Fig. 2), which brought clarity to the complex ‘current state’ patient journey. An IPO process map is a visual representation of a process, frequently used in multifaceted or complex processes to facilitate identification of critical process steps required to facilitate inputs evolving to outputs [24].

### Choice of solution

#### Analyse

Through the utilisation of a series of root cause analysis tools and approaches, the analyse phase allows teams to identify key areas critical to improving the ‘current state’ process. This analysis was essential to understand the reasons why the ‘current state’ was sub-optimal for patients and the hospital [25].

**Figure 3:**
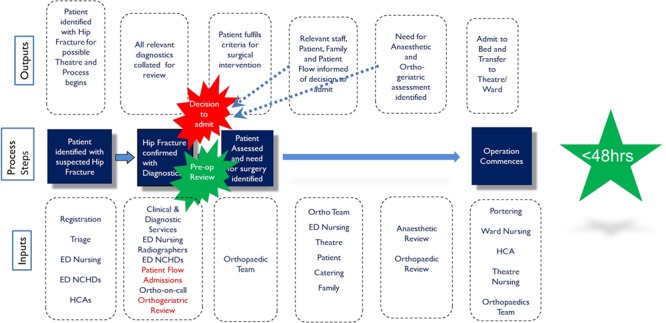
Analyse phase IPO process map depicting ‘future state’ hip fracture pathway.

The IPO process map was the key connection between the measure and the analyse phases. The IPO process map allowed the project team members to visually recognize the prime process steps that needed streamlining to improve clinical pathway efficiency and assisted the team in forming and visually illustrating the ‘future state’ process concept (Fig. 3).

The Lean tools of ‘silent brain storming’ and ‘five why’ were used to clarify the contributory causes to the identified process inefficiencies, and to identify possible solutions [18]. A ‘PICK chart’, an acknowledged LSS tool to assist prioritisation of concepts and solutions, was used to further analyse and refine these solutions to discern those that were both feasible and potentially impactful [26].

A key observation was that the waiting time for a surgical slot in theatre was prolonged, as it could only be requested once an inpatient bed had been allocated to each individual patient. Admission, and hence bed allocation, however, was requested only after patients were reviewed by an orthopaedic physician. A second observation was that there was no defined pre-surgery referral pathway to the orthogeriatric service for patients presenting with hip fracture. Patients that would potentially benefit from orthogeriatric input were identified on an *ad hoc* basis by the orthogeriatric team by reviewing admission details. This was considered, on discussion with the relevant stakeholders, to be both inefficient and ineffective. Additionally, it was recognized by the relevant stakeholders that fasica iliaca nerve block was within the scope of ED and orthogeriatric staff members, but no consistent training in this procedure was provided to staff, and so, an essential skill deficit was identified.

### Implementation

#### Improve

The improve phase of the project is focused on solution approach and developing an action plan for implementation [24]. The ‘future state’ IPO map (Fig. 3) illustrates two key process changes that were implemented in the improve phase to augment efficiency and streamline the patient journey from presentation to ED to surgery for hip fracture. A fast-track admission protocol was developed through consultation with ED medical and nurse management staff, patient flow, who are responsible for bed management, and the relevant orthopaedic consultants. This new admission protocol allowed the decision to admit and an inpatient bed to be requested by a clinical nurse manager in ED once hip fracture had been identified on X-ray. This eliminated the delay in admission previously engendered by having to wait for an orthopaedic review and then an orthopaedic surgeon to request an inpatient bed. Second, a formal referral pathway to the orthogeriatric service was agreed between ED staff and the orthogeriatric medical team. All patients ≥65 years with identified hip fracture were referred to the service by ED staff at the same time that these patients were referred for orthopaedic consult. This allowed early medical stabilisation of patients, again addressing an identified cause of delayed surgery.

To optimize analgesia provision, training of ED advanced nurse practitioners and orthogeriatric registrars in the fascia iliaca nerve block procedure was facilitated. This training was delivered by the Department of Anaesthesia and ED consultants. The implementation of these three strategies was achieved through regular engagement with the key stakeholders. These strategies are in line with Lean methodology, as utilising employee skills is considered important in reducing waste; employees are empowered and educated to work to their ‘top of licence’, thereby facilitating improvements being driven at local level [13].

#### Control

Sustainability of a project is key to the overall implementation [19]. This was supported with a robust control plan collaboratively agreed by the team and stakeholders. Such staff engagement facilitates staff to be decision makers in change implementation, further supporting project sustainability [ 27]. A communication plan was devised to ensure that all staffs involved in hip fracture care within the organisation were informed of the pathway improvements implemented with the aim of further enhancing staff engagement.

Continuous, prospective data monitoring is central to the control phase and defined staffs were assigned to this role with monthly dissemination of the performance metrics to the key stakeholders [14]. Furthermore, barriers to the maintenance of the implemented improvements were identified, with particular regard to the risks associated with regular changes of personnel in medical teams and wider hospital staff.

## Results

The three identified key performance metrics were assessed at 3 and 6 months following the implementation of the new initiatives identified in the improve phase, and compared to the retrospective data from 2014.

**Figure 4:**
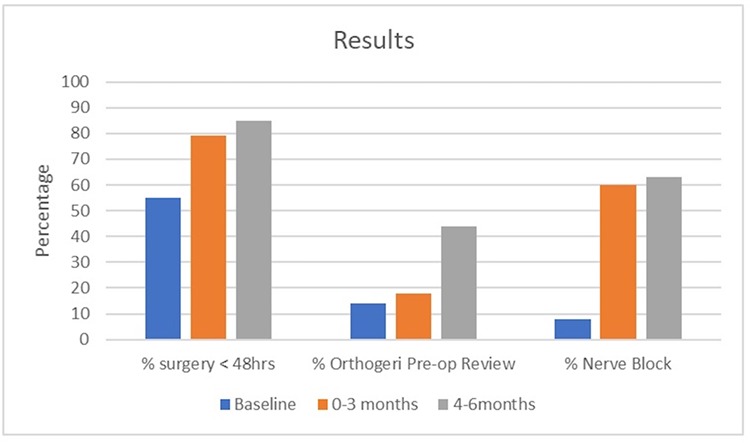
Graphic illustration of prime outcome measures, expressed as a percentage of total number of hip fracture patients ≥65 years, at baseline, 0–3 months and 4–6 months post-process improvements.

In 2014 nationally, 70% of patients ≥65 years with hip fracture underwent surgery within 48 h of presentation to ED; the corresponding figure for the project hospital was 55%. Data for the first 3 months post implementation revealed that this percentage had increased to 79%, and for months 4–6 post implementation, the proportion had further increased to 85%. This improvement is illustrated in Figure 4.

Figure 4 also outlines that in 2014 just 14% of hip fracture patients ≥65 years presenting to the hospital received pre-operative review from the orthogeriatric service; nationally in 2014, the figure was 7%. Three months post implementation, 18% of hospital hip fracture patients ≥65 years were reviewed pre-operatively by the orthogeriatric service. This percentage increased to 44% in the 4–6 months post-implementation period.

The 2014 national data revealed 19% of hip fracture patients ≥65 years received a fascia iliac nerve block in that year; the hospital’s performance for 2014 was 8%. As seen in Figure 4, after 3 months of implementing the strategies identified by the LSS DMAIC process, this proportion markedly increased to 60%, and remained consistent for the duration of the study period, with 63% receiving nerve blocks between months 4 and 6.

## Discussion

This study contributes to the growing body of published evidence that LSS methodology that originated in the manufacturing sector can be successfully employed to optimize care and resource use in the health sector and improve patient flow, through a focus on process redesign, enhanced teamwork and role redistribution, but with minimal cost implications to the organisation.

Patient flow is defined as the movement of patients, information or equipment between departments, staff groups or organisations as part of a patient’s care pathway and corresponds to flow in Lean manufacturing [21]. Hellstrom *et al*. [28] highlighted that flow orientation in healthcare systems is often inadequate. In healthcare, flow orientation is challenged by complex care processes that involve multiple healthcare units or teams; when multiple teams are involved, individual staff members frequently do not experience the entirety of the patient journey or the work process, and it may be difficult to identify who should take responsibility for the complete patient flow [11].

Co-ordination and integration of care is one of the identified dimensions of patient-centred care and is integral to efficient patient flow [29]. Staff engagement and improved inter-team working were integral to the success of the process redesign; specifically, communication and co-ordination of care between the ED, orthopaedic, orthogeriatric and anaesthetic teams were improved, resulting in a more streamlined and more integrated care pathway. Although each of the four individual teams involved in delivering hip fracture care in the hospital may have worked well in and of themselves, the broader vision of the four teams together forming a single larger team in the delivery of hip fracture care was absent. Each individual team worked to deliver their segment of the care pathway, but there was limited communication, and hence integration of care across the teams.

The challenge of developing effective teamwork in hospitals is acknowledged in the literature, as hospitals have both a hierarchical structure and independent professional groups with deep-rooted stand points on scope of practise [30]. Facilitating the convergence of the four medical teams in pursuit of the defined and unified goal of ensuring that more patients presenting with hip fracture undergo surgery within 48 h of presentation was one of the biggest challenges encountered by the project team. The project team aimed to facilitate a meeting with staff from each of the four teams to facilitate consensus and an agreed path forward for solution implementation. This, disappointingly, was not achieved due to difficulty in co-ordinating the schedules of busy healthcare professionals. Instead communication was primarily facilitated by project team members on an individual basis with each team and with all teams via email. Perhaps a single meeting with the key stakeholders from each medical team, as originally planned, would have achieved similar, or even enhanced, improvements with less effort and time commitment from the project team members.

Nevertheless the inter-team communication facilitated by the project team engendered tangible changes. Task redistribution in the interest of enhanced patient care was facilitated; the established practise of orthopaedic physicians requesting inpatient admission for hip fracture patients was discarded, and reassigned to the ED CNM to facilitate the earlier initiation of this essential process step.

Facilitating inter-team discussion resulted in the additional boon of addressing optimal patient analgesia for hip fracture patients. This was not an original project goal identified in the Project Charter, but was added when identified by the Department of Anaesthesia as a person-centred performance metric that expedites patient care in theatre, and enhances quality care, patient comfort and patient experience. Facilitating training in the fascia iliaca nerve block technique brought interdisciplinary team members together, with the Department of Anaesthesia hosting training for ED nursing and medical staff, and orthogeriatric physicians, with benefits of role diversification facilitating care from ‘the right person at the right time’ and enhanced team working and communication.

Aij *et al*. [31] highlighted insufficient available time as a barrier to successful Lean implementation in healthcare, including the release of staff from their other commitments to complete project work. In this project, the time cost was not recorded or evaluated. It is acknowledged that such information would be very useful in the planning of future projects. Certain tasks were very time-consuming, particularly completing the real time Gemba visit. Other aspects, considered to be very important to the final outcome, were not costly in terms of staffing resources; for example, the data collection required for the performance metrics was already in place. The addition of monthly dissemination of this information was considered an important, and to date an on-going, step to aid maintenance of results through keeping the project aims and outcomes in the consciousness of the key stakeholders with minimal time cost to the data collators. No project team input was required in months 3–6 post implementation; thus, performance maintenance for that period attests to the project’s sustainability and the success of the control phase.

Although the proportion of patients receiving pre-surgical orthogeriatric review tripled over the duration of the study, the end of study percentage did not exceed 50%. This may be explained in part by the orthogeriatric team, despite receiving a referral, not being able to assess patients pre-operatively due to caseload prioritisation or by having insufficient time pre-surgery, especially for referrals generated over weekends and outside of normal working hours. Further study is needed to elucidate the reasons for the low percentage of patients receiving pre-surgical orthogeriatric review, as well as potential consequences for patient care.

This study took place in a large, acute Irish hospital that has a Lean Academy supported by hospital management. The Lean Academy has been training staff in the LSS approach and has been involved in hospital service improvement projects for some years. Extrapolating the success of this project to different healthcare contexts in different jurisdictions and in the absence of an organisational understanding of LSS methodologies is ill advised. Nevertheless, this study demonstrates that LSS methodology can be easily and successfully implemented in the healthcare environment, facilitating effective team working and enhanced patient care through process improvement.

## Conclusions

The aim of the project was to improve acute hip fracture care. The adoption of LSS methodologies as an approach to continuous improvement within the hospital assisted in attaining improvements within the acute care setting, thereby advancing patient care and the hospital’s performance in key, nationally monitored standards in hip fracture care, but crucially without a cost implication for the hospital. Process redesign with enhanced teamwork and role redistribution were central to this project’s achievements. Continued success of the outcomes will require frequent monitoring and adaption of the process as the new pathway embeds and evolves in practise.
